# Supplementary material for: Endothelial junctional membrane protrusions serve as hotspots for neutrophil transmigration
Source: eLife. 2021 Aug 25;10:e66074. doi: 10.7554/eLife.66074 (PMC8437435; doi:10.7554/eLife.66074)
Supplement: Figure 4—source data 1. [file elife-66074-fig4-data1.pdf]

Data source: Western blotting

Figure 4
Supplemental Figure 4B

Supplemental Figure 4E

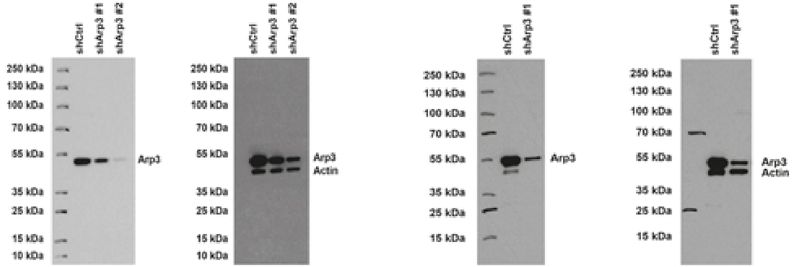

Source Data Western blotting Arts et al. **Figure 4 Supplemental figure 4B:** HUVEC samples were analysed for Arp 3 knock down. Samples were lysed and treated as described in Methods. Western blots were stained for Arp3 primary antibodies and secondary HRP labeled antibodies for chemiluminescence. Full blots are displayed.
