# Supplementary material for: Endothelial junctional membrane protrusions serve as hotspots for neutrophil transmigration
Source: eLife. 2021 Aug 25;10:e66074. doi: 10.7554/eLife.66074 (PMC8437435; doi:10.7554/eLife.66074)
Supplement: Figure 6—source data 1. [file elife-66074-fig6-data1.pdf]

Figure 6

Supplemental Figure 6E:

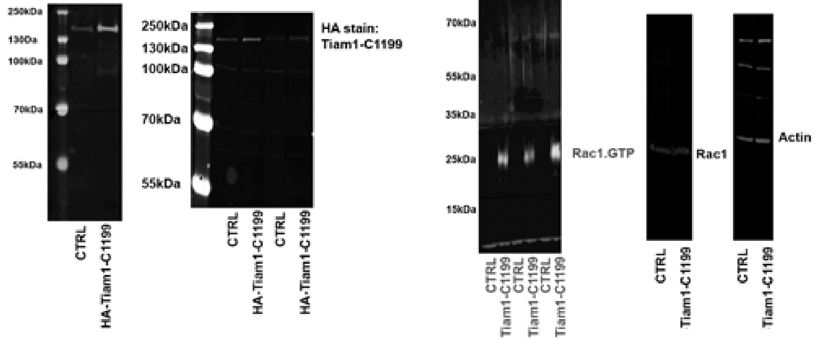

Source Data Western blotting Arts et al. **Figure 6 supplemental figure 6E**: HUVEC samples were analyzed for Rac1 activity when transfected with Tiam1-C1199. Transfection of HA-Tiam1-C1199 constructs were checked on expression by Western blotting and staining with primary HA antibodies, followed by detection with IRDye 800CW or IRDye 680RD secondary antibodies. Detection was done using an Odyssey developer.
